# Resilience enabling processes and posttraumatic growth outcomes in a group of women survivors of childhood sexual abuse

**DOI:** 10.4102/hsag.v23i0.1134

**Published:** 2018-11-28

**Authors:** Hayley J. Walker-Williams, Ansie Fouché

**Affiliations:** 1 School of Psychosocial Health, Faculty of Health Sciences, Optentia Research Focus Area, North-West University, South Africa

## Abstract

**Background:**

This study forms part of a pilot project, aiming to evaluate the benefits of a programme entitled S2T denoting from Survivor to Thriver, which is a collaborative strengths-based group intervention programme for women survivors of childhood sexual abuse. The objective of the S2T is to enable resilience processes and facilitate posttraumatic growth outcomes. A theory of change was developed to explain how these objectives are met, it outlines the theoretical approach, mediators, primary and secondary outcomes.

**Aim:**

To explore emerging resilience processes and posttraumatic growth outcomes in women survivors of childhood sexual abuse after attending the S2T.

**Setting:**

Data was collected during group treatment sessions of the S2T group intervention programme with women survivors of childhood sexual abuse.

**Methods:**

A quasi-experimental, one group, pretest, posttest, time-delay design was used with eight purposively selected women with a history of childhood sexual abuse, using qualitative methods to evaluate the benefit of this intervention. Nine group treatment sessions and a delayed follow-up session were conducted. A visual participatory technique and transcriptions of group sessions were used to collect qualitative data.

**Results:**

Qualitative thematic analysis revealed the following enabling resilience processes: group as healing vehicle of change; changing destructive to constructive rumination; coping strengths and meaning-making. Posttraumatic growth outcomes which evolved were: transforming wounded to healer; a changed life philosophy; excavated spirituality and re-briefing strengths through a posttrauma thriver identity.

**Conclusions:**

The themes suggest resilience processes and posttraumatic growth outcomes; a longitudinal study is recommended to establish effectiveness and inform treatment practice.

## Introduction and background

A recent systematic review of meta-analyses on the incidence of child maltreatment indicates a global prevalence in self-reports of childhood sexual abuse (CSA) at 18% for girls and 7.6% for boys (Stoltenborgh et al. 2015). The first national representative study on the prevalence of CSA in South Africa indicates that one in every three young people has experienced some form of sexual abuse, and that the prevalence amongst boys is 36.8% and 33.9% in girls (UBS Optimus Foundation 2016).

Some authors argue that should CSA be left untreated, it could lead to mental health, sexual and inter- and intrapersonal difficulties (Finkelhor 1990; Mathews, Abrahams & Jewkes 2013). In addition, Finkelhor and Browne (1985) report on specific trauma factors in the CSA context, which delineates this trauma as unique, namely traumatic sexualisation, betrayal, stigmatisation and powerlessness. These trauma-causing factors – also referred to as traumagenic dynamics – might result in the development of negative trauma messages internalised by the survivor with regard to themselves, others and the world, and may end in several short- or long-term negative or pathogenic psychological and behavioural manifestations thus making the rendering of effective treatment to women survivors of CSA imperative.

To date, the dominant approaches in CSA treatment have focused on the traditional pathogenic or deficit paradigm. Within this paradigm, the focus is on symptom reduction and returning the survivor to baseline functioning (Taylor & Harvey 2010). This approach is based on the medical model and regards survivors as problem-ridden, powerless and in need of repair. It thus disregards their intrinsic or extrinsic resources available to aid in their recovery (Cummins, Sevel & Pedrick 2014). These traditional approaches used in treating survivors of CSA are inter alia cognitive behavioural therapy (CBT) (Wilen, Littell & Salanti 2012), psychodynamic therapy (Lord 2008) and psychoeducation (Brown et al. 2013).

The strengths-based approach is challenging this dominant traditional approach with the question: ‘Is it only what’s wrong, or also about what’s strong?’ In doing so, the strengths-based approach advocates for the sourcing of intrinsic or extrinsic resources available to aid in the survivors’ recovery (Teater 2014), while not discounting the negative impact. Similarly, both authors, who have vast experience in working with survivors of CSA, pondered such questions as: Can strengths be borne out of some women’s CSA struggle? Can recovery be an opportunity for growth where the survivor becomes the ‘expert’ of her own healing? Can the survivor’s social ecology facilitate recovery? This reflective process ties in with an international call for a salutogenic approach in treating communities of vulnerable women survivors (Walker-Williams & Fouché 2017).

Treatment of CSA survivors could be approached individually or in group contexts. Literature is scarce on differentiating between the effectiveness of individual and group treatment. However, Kessler, White and Nelson’s (2003) systematic review found group treatment to be the most effective treatment in the recovery of women survivors. Given the scarcity of therapeutic CSA intervention resources in South Africa, and the effectiveness of group treatment in reducing cost and stigmatisation in such survivors (Brown et al. 2013), we adopted a group treatment approach. As such, we developed a collaborative strengths-based group intervention, entitled S2T (denoting from Survivor to Thriver), for women who experienced CSA, integrating elements of traditional CSA treatment approaches with a strengths-based approach. To date, the S2T development and pilot testing have focused on three groups of women. The groups were not implemented concurrently but in succession. The first group included 10 women and commenced in 2013 and ended in 2014. The findings of this pilot group were reported by Walker-Williams and Fouché (2017). The findings of the second pilot group, which included eight women and commenced in 2014 and ended in 2015, will be reported in this study. The third group comprising five women commenced in 2017 and will be concluded towards the end of 2018. The recruitment procedure will be discussed under research methodology. This study thus aims to report on the emerging resilience-enabling processes and posttraumatic growth outcomes of the second group of women who attended the S2T collaborative strengths-based group intervention programme.

Next, the theoretical foundation of the S2T will be explained whereafter the methodology, findings and the way forward is discussed.

## Theoretical approach

The S2T collaborative strengths-based group intervention programme for women survivors of CSA is theoretically grounded in – and draws on – traditional CSA treatment approaches, namely CBT (Wilen et al. 2012), psychodynamic therapy (Lord 2008) and psychoeducation (Brown et al. 2013); an integrated trauma model, the Wits Trauma Model (Eagle 2000) and a strengths-based approach.

A strengths-based approach is defined as using a:

wide range of practice principles, ideas, skills and techniques to promote and draw out the resources of clients and those in the environment so as to initiate, energise and sustain change (Cummins, Sevel & Pedrick 2012:51).

The strengths-based approach focuses on the person’s strengths and how these may be used as resources for change. As such, supportive ecologies are seen as enabling resources towards coping with abuse histories (Saleeby 2002). Within the strengths-based perspective, several models are observed in practice, inter alia resilience (Orbke & Smith 2013; Saleeby 2002) and the posttraumatic growth model (Tedeschi 2010; Tedeschi & Calhoun 2004, 2006).

Globally, there exists no universal definition of resilience. However, two key elements reflected in the resilience literature (Domhardt et al. 2015; Marriott, Hamilton-Giachritsis & Harrop 2013; Orbke & Smith 2013) are, first, that resilience occurs in the context of adversity, such as the exposure to a trauma (e.g. CSA), and second, positive adaptation, which entails the enabling processes following such exposure. In the context of CSA, authors refer to resilience as competence in the face of adversity (Hyman & Williams 2001) and adaptive functioning (Domhardt et al. 2015) or someone who maintained well-being despite having experienced intense trauma (Newsom & Myers-Bowman 2017).

A body of research has explored resilience processes in adult survivors of CSA. As such a recent systematic review including 37 studies found various individual and environmental resilience processes that contributed to CSA survivors’ adaptive functioning. These were education; psychoeducation; emotional and interpersonal competence; optimism, hope and control; belief in their own future; attachment to family and peers; and social support (Domhardt et al. 2015). Earlier researchers, Hyman and Williams (2001), in an empirical study of 136 women found six protective resilience processes in women survivors of CSA. These were growing up in a stable family; perpetrator not being a family member; absence of physical force during the CSA; not being arrested when young; graduating from high school and not experiencing revictimisation as an adult. In a small qualitative study, six women survivors of CSA reported six meaningful outcomes in their recovery journey: recognising that CSA does not define you; letting go of negative emotions; letting go of negative thoughts; developing healthy intimate relationships; accepting yourself as a sexual being; and finding and engaging in a healthy sexual relationship (Hartley et al. 2016).

It, thus, appears that important resilience processes on an individual level are education, believing that one can influence one’s surroundings and believing that one can learn and grow from life experiences. In addition, the role of the ecology as an enabling factor in the face of adapting to the CSA should be acknowledged (Domhardt et al. 2015; Kaye-Tzadok & Davidson-Arad 2016; Marriott et al. 2014; Ungar 2017).

Posttraumatic growth is defined as positive psychological change experienced as a result of the struggle with highly challenging life crises (Tedeschi & Calhoun 1996, 2006). It is seen as a multidimensional construct that involves transformational changes divided into three general domains: changes in the experience of relationships (improved relationships); changes in perceptions of self (enhanced personal strengths and new possibilities in life) and changes in one’s life philosophy or outlook on life (greater appreciation for life and spiritual change). Several empirical studies confirm that it is possible within the struggle of the CSA traumatic ordeal for survivors to experience posttraumatic growth (Hartley et al. 2016; Lev-Wiesel, Amir & Besser 2004; Shakespeare-Finch & de Dassel 2009). In a small qualitative study of six female survivors of CSA, three posttraumatic growth outcomes were found: making sense of and understanding the CSA in relation to growth; relating to oneself in a novel way and acknowledging positive outcomes and experiencing growth post trauma through engaging in relationships with others (Hartley et al. 2016). A quantitative study including 100 women survivors found that cognitive strategies and family support were outcomes of posttraumatic growth (Kaye-Tzadok & Davidson-Arad 2016).

For the purposes of this study, resilience is seen as a process of positive adjustment despite exposure to CSA adversity by drawing on internal and external resources. In addition, posttraumatic growth can be seen as experiences of positive psychological growth emerging post trauma in relation to the self, others and the world.

### Development and core components of Survivor to Thriver

When developing a group intervention, several aspects need to be considered, such as the existence of a theory of change (Box 1); clearly defined and considered inclusion and exclusion criteria; prior decisions regarding the handling of attrition and the reporting of participant numbers; selection of an appropriate study design; the use of sound evaluation methods; ethical considerations and a standard of care. Lastly, the treatment should be manual-based, and measures should be in place to ensure that the facilitator adheres to the treatment manual (Kessler et al. 2003).

BOX 1 Survivor to Thriver theory of change.

**Theoretical approach**

- Empirical study developed for South African context
- Eclectic mix of theories (CBT, psychodynamic, psychoeducation)
- Salutogenic (strengths-based) paradigm
- Resilience and posttraumatic growth model

**Mediators**

- Provide safe contained space
- Share trauma story and messages
- Engage in constructive rumination
- Use decisive action/internal locus of control
- Improve social cohesion
- Meaning-making and benefit-finding and expert companion and witnessing

**Secondary outcomes**

- Generate a healing group context
- Increased introspection /emotional awareness
- Cognitive reframing and restructuring
- Increase resilient, driven, adaptive coping
- Build social support strengths
- Develop a posttrauma narrative

**Primary outcomes**

- Posttrauma thriver identity
- Personal strengths
- Improved personal relationships
- Greater appreciation for life
- New life possibilities
- Deepened spiritual development

CBT, cognitive behavioural therapy.

The S2T was developed from an empirical study, including a quantitative study (in which 60 women CSA survivors completed standardised instruments measuring their posttraumatic growth, psychological well-being and coping attempts) (Walker-Williams, Van Eeden & Van der Merwe 2012); a qualitative component (in-depth interviews with 10 women displaying posttraumatic growth and adaptive coping) (Walker-Williams, Van Eeden & Van der Merwe 2013); a literature review and practice experience of both authors (a clinical psychologist with 16 years’ experience, and a social worker with 24 years’ experience) (Fouché & Walker-Williams 2016).

As depicted in Table 1, the S2T comprises an eclectic mix of traditional approaches (CBT, psychodynamic and psychoeducation), an integrated trauma model (the Wits Trauma Model) and the strengths-based models of resilience and posttraumatic growth. The S2T programme consists of six treatment outcomes that follow the progression of victim to survivor to thriver narrative roles. The process is not linear, and requires constant circular reflection. The group sessions can include up to nine sessions, each with distinct objectives, activities, narrative roles and outcomes.

**TABLE 1:** Summary of Survivor to Thriver (S2T) treatment outcomes, objectives and activities.

S2T treatment outcomes, objectives, and narrative role	Activities and facilitation techniques	Theoretical approach
*Outcome 1:* Providing a healing group context *Objectives:* Supportive, structured, contained and accepting environment facilitated by expert companions	Gaining informed consent, clarifying roles and expectations, setting group commitments and encouraging confidentiality Safe word assigned to indicate feelings of being unsafe or uncontained Group metaphor of healing chosen to indicate the group’s unique common identity Facilitation by competent facilitators as expert companions Focus on Saleeby’s (2002) framework of questions and probes to assess strengths	Psychodynamic Resilience /posttraumatic growth Wits Trauma Model
*Outcome 2:* Introspection and heightened emotional awareness *Objectives:* Telling the trauma story *Narrative role:* Victim	Draw-and-write and draw-and-talk activity (Mitchell et al. 2011) Explore facts, feelings, cognitions and sensations during the childhood sexual abuse (Eagle 2000) Explore support at the time of the childhood sexual abuse and their subjective opinion on how it has affected their identity and psychosocial functioning to date (Stockton, Hunt & Joseph 2011; Tedeschi 2010) Facilitators to contain and process strong emotional reactions	Cognitive behavioural therapy Psychodynamic Wits Trauma Model
*Outcome 3:* Cognitive processing and restructuring *Objectives:* Identify and explore internalisations, normalise symptoms, deal with loss and reframe internalisations *Narrative role:* Survivor	Normalise symptoms (Eagle 2000) Psychoeducation on trauma-causing factors (Finkelhor & Browne 1985) Address internalisations (Fouché & Yssel 2006) Focus on cognitive distortions (Fouché & Yssel 2006) Internalisation and boundary activities, for example glasses, robot and egg analogies (Fouché 2006; Fouché & Yssel 2006; Fouché & Walker-Williams 2016) Self-nurturing techniques Metaphorical burning ritual Letter to perpetrator Explore the stages of loss and the role of forgiveness	Cognitive behavioural therapy Psychodynamic Psychoeducation Wits Trauma Model
*Outcome 4:* Active adaptive coping *Objective:* Decisive action and using an internal locus of control *Narrative role:* Survivor	Explore current coping repertoires Build constructive coping tool boxes, for example strong foot (Walker-Williams et al. 2012) Self-esteem activities Self-nurturing techniques (Walker-Williams et al. 2012) Growth journal Positive affirmations	Psychoeducation Strengths perspective Resilience Wits Trauma Model
*Outcome 5:* Social support strengths *Objective:* Connecting with family, friends and significant others *Narrative role:* Survivor	Build action plans for positive connections in relationships Gratitude journal	Strengths perspective Resilience/posttraumatic growth
*Outcome 6:* Posttrauma identity *Objective:* Meaning-making and benefit-finding (strengths emerging from struggle) *Narrative role:* Thriver	Strengths building Value in Actions Questionnaire (Compton 2005) Retelling the story for a ‘change’ (Walker-Williams et al. 2012) Reinforce behaviours, thoughts or strategies indicative of mastery in trauma experience (Eagle 2000) Visual participatory method (draw-and-talk and draw-and-write) (Mitchell et al. 2011) Comparison of drawings before and after intervention (Walker-Williams & Fouché 2017) Explore post-trauma identity (combining meaning-making and benefit-finding) Group narrative (Walker-Williams & Fouché 2017) Congratulatory thriver ceremony (Walker-Williams et al. 2012)	Strengths perspective Resilience/posttraumatic growth

Three important mediators included are the group setting, regarded as the vehicle of change; the value of ‘witnessing’, when group members vicariously experience each other’s stories and lastly the facilitators who act as ‘expert companions’ in navigating the CSA survivors, still regarded as the expert of their own trauma (Tedeschi & Calhoun 2006:292). The stance of the facilitator as expert companion requires an ability to perceive subtle indications of growth and recovery where the client’s trauma experiences are recognised as potential sources of learning, rather than merely a collection of symptoms to be remedied (Tedeschi & Calhoun 2006).

Once the theory of change was constructed, the treatment manual was developed, together with a process evaluation form that was completed after each session to ensure protocol adherence.

## Research methodology

### Research design

We used a quasi-experimental, pretest–posttest, one group only, interrupted time series design (Leedy & Ormrod 2005; Rubin & Babbie 2016). Due to the stigma and secrecy surrounding CSA, access to this vulnerable population is restricted. Consequently, the authors were unable to recruit enough women to randomly select them into an experimental and comparison group, thus a quasi-experimental design was most suited to this research study (Yegidis, Weinbach & Myers 2012). The S2T programme spanned a 3-month period and nine group-facilitated sessions took place. The group sessions typically lasted between 2 to 3 hours, and were conducted at 2-week intervals. A delayed follow-up session was conducted 9 months after the ninth session during which a second posttest was administered.

### Participants and sampling

Purposive sampling was used, and participants were recruited via psychologists and social workers in the Vaal Triangle area who acted as gatekeepers, using the following selection criteria: CSA before the age of 18 years, the abuse had been disclosed; individual therapy had taken place; and there was a need for further intervention. Eight women commenced (five black and three white) and five (three black and two white) completed the group treatment. Ages ranged between 18 and 36 years and the average age was 25 years. The five women who completed the programme were between the ages of 20 and 36 years with an average age of 26 years. All women experienced contact abuse during middle childhood, by a known male perpetrator (in most cases intrafamilial). No criminal cases were opened.

### Evaluation methods

We used qualitative evaluation methods during the pre- and two posttests. A visual participatory approach was used using the draw-and-talk and draw-and-write techniques (Mitchell et al. 2011). According to Mitchell et al. (2011), drawings can assist adults in capturing memories, thoughts and feelings that are not easily transformed into words. The following instruction was given during the pre-, post- and delayed posttest (during session 1, 9 and the delayed session, respectively):

Draw a symbol of how you have coped with your traumatic CSA ordeal and what growth (if any) you have experienced as a victim of this childhood trauma? Then, explain in writing (e.g. write down a couple of sentences) on what your drawing is saying about how you have coped and grown (if at all) from your CSA experience.

To prevent any subjective interpretation by the researchers, participants were requested to compare and interpret their first, second and third drawings and share these as well as their narratives. In addition, the transcripts of the treatment sessions were also analysed to identify emerging resilience processes and posttraumatic growth outcomes.

### Data analysis

In addition to the drawings and narratives by the participants, transcriptions of the audiotaped sessions were analysed. Both authors and an independent coder conducted thematic analysis of the transcribed treatment sessions as well as the pre- and post-test narratives, and met for consensus discussions, after which themes and subthemes were established.

### Trustworthiness

The trustworthiness of the qualitative data can be enhanced using four processes, namely credibility, transferability, dependability and conformability (Denzin & Lincoln 2001). The extent to which the data and data analysis are believable and trustworthy is called credibility. Because of the dual role of the authors as researchers and group facilitators, a reflexive approach was followed (Trondsen & Sandaunet 2009). An observer attended all group sessions to evaluate adherence to the protocol. We further engaged in member-checking (participants interpreted their own drawings), made use of an independent coder and several rounds of consensus discussions, as well as a research journal (Arber 2006). Before and after sessions, the researchers spent time reflecting on the participants’ individual and group processes. Denzin and Lincoln (2001) describe the extent to which other researchers can apply the findings of the study to their own contexts as transferability. In this regard, the S2T intervention is manual-based, and is guided by an intervention protocol providing in-depth instructions regarding the implementation and facilitation (Shenton 2004). Confirmability is the extent to which the research findings can be corroborated by others, and dependability is the extent to which research findings can be replicated with similar participants in similar contexts (Denzin & Lincoln 2001). In this regard, member-checking was used, and participants interpreted and analysed their own drawings and narratives. Three group members dropped out after the first and second group sessions, respectively. The first group member preferred to continue individually, and the first author continued seeing her separately, until the group member terminated the contract. The other two group members seemed reluctant to continue exploring their CSA experiences in a group context. Attempts were made to encourage them to return to the group; however, these endeavours proved unsuccessful.

### Ethical considerations

Ethical clearance was obtained from the University’s Ethics Committee before commencement of the intervention (NWU-00041-08-A1). Participants were provided with adequate information about the research study, so as to enable them to make an informed participatory choice. In this regard, participants were issued an introductory letter outlining information pertaining to the study, accompanied by an informed consent form that was then signed, as proof of voluntary participation. Confidentiality assurance and the preservation of dignity were provided during the intervention and also throughout the research. The voice recordings and transcriptions were securely stored after completion of the data analysis (Creswell 2014). The S2T intervention was manual-based and guided by an intervention protocol that provided in-depth instructions on the implementation and facilitation of the S2T intervention programme (Shenton 2004). Facilitators offered extra support outside the group, and access to counselling services was available as required.

## Research findings

In Table 2, the four resilient enabling processes and four posttraumatic growth outcomes are depicted. The findings will now be presented by discussing the emergent themes, using extracts from the participant’s narratives in the form of verbatim quotations from the transcripts. Numbers were assigned to each participant.

**TABLE 2:** Resilience enabling processes and posttraumatic growth outcomes.

Resilience enabling processes (I CAN)	Posttraumatic growth outcomes (I AM)
**Group as healing vehicle of change** Normalising space Witnessed by other survivors	**Transforming wounded to healer** Need to help others Giving unconditionally
**Changing destructive to constructive rumination** Self-awareness Cognitive reframing and restructuring	**Changed life philosophy** Re-evaluation of self Future perspective Greater appreciation for life
**Coping strengths** Experimenting with changed behaviour Engaging in social support	**Excavated spirituality** Faith as a source of strength Comfort through relationship with God
**Meaning-making** Acceptance and purpose Religious activities	**Re-briefing strengths through post-trauma thriver identity** Painful sharpenings make a better pencil Acknowledging the new ‘me’

### Resilience enabling processes

#### Group as healing vehicle of change

Participants reported finding the group setting a normalising environment where they felt free to open up and share their CSA experiences after witnessing (hearing) the stories of fellow group participants. This safe holding environment seems to have become a vehicle of change, as is illustrated in the following themes.

**Normalising space:** Participants 2 and 4 highlighted that the group was a safe normalising space where change could occur, as illustrated in the following comments:

‘I think coming and speaking and hearing other people have problems too, and just some advice that you give to others has helped me to skip through the external things.’ (Participant 2, female, 36 years old)

‘When you hear other people’s stories, it just makes you think that you know what, it could have been worse, and you feel that, I don’t know, that sense of sympathy for another person, you feel for them so much …’ (Participant 4, female, 22 years old)

**Witnessed by other survivors:** In the following extract, participant 3 reflects on how she was inspired to get stronger by a fellow survivor in the healing group space: ‘I find that you [*participant 5*] are a very strong person and I would like to be more strong like you …’ (Participant 3, female, 18 years old)

#### Changing destructive to constructive rumination

Participants reported a very specific, enabling process pertaining to their cognitive appraisal of the CSA trauma which involved heightened self-awareness, and applying new knowledge gained in order to engage in cognitive reframing and restructuring, as illustrated in the following subthemes.

**Self-awareness:** Participants 1 and 5 highlighted that they were able to reflect on their own awareness of how their lives had transformed, resulting in them becoming more self-accepting. This apparently led to higher levels of introspection pertaining to the impact of the CSA trauma on their emotional and overall functioning:

‘My whole perspective in life changed … it was a secret and then suddenly … that lie is gone, I don’t have to perform, it’s not a race … so I’m just me … and it’s so nice …’ (Participant 1, female, 30 years old)

‘I think I’m starting to realise that they are at fault in my life, and how I’m blaming myself for certain things that I shouldn’t.’ (Participant 5, female, 20 years old)

**Cognitive reframing and restructuring:** The following participants indicated how the understanding and application of new knowledge gained in the group initiated the process of cognitive reframing and restructuring. This was a constant process of devotion as seen in the following extracts by participants 1 and 2:

‘… side and side there is no magic that will take the pain away, you have to work on it every day.’ (Participant 1, female, 30 years old)

‘… in the end I also had to make the conscious choice, it wasn’t just about me, and I had to make the choice, do I want to live or do I want to die because if I want to live I’ve got to get my butt up in the mornings, no matter how difficult it is … am I going to live or am I going to die?’ (Participant 2, female, 36 years old)

#### Coping strengths

Participants reported two specific enabling processes in developing coping strengths. These were experimenting with changed behaviour and engaging in social support, as illustrated in the following subthemes.

#### Experimenting with changed behaviour

Participants 1 and 6 shared that emotional self-regulation, engaging in positive activities, exercising and having a routine are key to adaptive coping outcomes:

‘… we need to keep in routine…and then exercise … and then to surround yourself with positive people and good people …’ (Participant 1, female, 30 years old)

‘… self-regulation is a process I have to work on … and yeah I think through this group we’ve all become more self-regulated, so it is something I’ve been working on.’ (Participant 6, female, 21 years old)

**Engaging in social support:** Participants 2 and 4 highlight the necessity to engage in positive reciprocal relationships that foster a value-laden relationship:

‘We have a very close relationship, our family is extremely close, and I wouldn’t trade my dad for anyone. I’d rather be fighting with him than not have him around at all … we did a lot of activities as a family together …’ (Participant 2, female, 36 years old)

‘I guess to be involved in more positive activities…to be with people who I love, feel comfortable with … add worth to my life.’ (Participant 4, female, 22 years old)

#### Meaning-making

Participants reported the importance of acceptance and having a purpose and spiritual connection so as to foster meaning of the CSA trauma, as illustrated in the following subthemes.

**Acceptance and purpose:** Participants 1 and 2 reflect on how assisting others could foster hope and meaning:

‘… and in that act of helping someone else to find hope, you find meaning … I don’t know, it’s a mystery, I don’t know exactly how to explain exactly how you find that meaning, but you do.’ (Participant 1, female, 30 years old)

‘Also what I need to change from the … victim to a survivor … I felt is personal acceptance, love and assurance, both from myself as well as others.’ (Participant 2, female, 36 years old)

**Religious activities:** In the following extracts, participants 2 and 4 reflect on the importance of prayer and religious conviction as a source of strength, as evidenced in the following comments:

‘I think what would drive me is … I pray, and prayer is not a one-way thing, prayer is a conversation that you have with God. And I mean the one night God spoke to me … and it was like this clear as daylight voice talking to me … and I think that talking to God has been my strength.’ (Participant 2, female, 36 years old)

‘I pray a lot … and there was a point where I even fasted … because God is not going to come in person, he is going to make a way and I know I’m good, I know … So I pray a lot … I pray because there’s no-one I can talk to outside of these walls.’ (Participant 4, female, 22 years old)

### Posttraumatic growth outcomes

#### Transforming wounded to healer

A specific turning point in the growth process of the participants was when they became aware of being focused on the needs and suffering of others instead of themselves, as evidenced in the following subthemes.

**Need to help others:** As illustrated by participants 1 and 2, suffering might well create an opportunity to assist others:

‘I think I found good in my suffering, and I said if my suffering oozes good things, by helping other people, then it will have been worth it.’ (Participant 1, female, 30 years old)

‘I had to choose life, because there are so many other people there that need me as well, they love me and [*I’ve*] got to realise that … it’s not always just about me … so getting up was to start living.’ (Participant 2, female, 36 years old)

**Giving unconditionally:** Participant 6 related to the need to give love unconditionally without the fear of rejection:

‘At first I thought that I was not going to be loved, but now I am worth being loved, and I am worth genuine love … and I believe that the kind of love that I can give is unconditional because I know that you do not give love in the circumstances.’ (Participant 6, female, 21 years old)

#### Changed life philosophy

Participants reported the importance of the re-evaluation of the self, and a purpose-driven life with a greater appreciation, as illustrated in the following subthemes.

**Re-evaluation of self:** In the following extracts, participants 1 and 5 reflect on the importance of challenging their views of self in relation to the CSA trauma:

‘… why am I the way that I am … it’s like an emotional disability…it’s like I’m in an emotional wheelchair … it’s something that’s always going to be there … it’s a process I understand, now I know I don’t need to feel insecure any more, I don’t have to think people don’t like me anymore, but I need to evaluate myself every time … it’s hard, it’s a long process, but it’s worth it.’ (Participant 5, female, 20 years old)

‘I never used to acknowledge it, but now I do. I acknowledge how I feel … I’m going to do what I can, what I’m capable of.’ (Participant 1, female, 30 years old)

**Future perspective:** Participant 2 acknowledged wanting to grow up:

‘… I want to be an adult … I want to grow up because I wasn’t allowed to grow up … so now I want to grow up.’ (Participant 2, female, 36 years old)

**Greater appreciation for life:** Participant 2 and 6 reflected on their appreciation of life and the need to create a better future:

‘for once I appreciated my life … I am in varsity, trying to build a better future for myself … for my family as well … I’m just a better person … so I am a thankful person … for every little thing that’s happening.’ (Participant 2, female, 36 years old)

‘I was not a happy child … and the minute I started seeing how other people were grateful for the little things, that’s when I saw that positivity changes everything … so for me I would say it was a coping mechanism … let me help somebody out, that way I will not feel what I’m feeling right now.’ (Participant 6, female, 21 years old)

#### Excavated spirituality

Participants reported that faith was a source of strength, and that a sense of comfort was gained through their deepened spiritual development, as illustrated in the following subthemes.

**Faith as a source of strength:** Participant 5 highlighted that her faith should remain a protective factor:

‘… my faith in God should stay and what should change are my thoughts regarding the situation … and I guess feelings of insecurities and people that bring me back to the point of feeling not good enough.’ (Participant 5, female, 20 years old)

**Comfort through relationship with God:** Participant 6 admitted to realising that her relationship with God has been a constant and is on a deeper unconscious level:

‘I’ve come to the realisation that God is not a God of solutions…He’s a constant God, so it doesn’t mean that even when things are not going my way, He’s not part of it … I’ve realised that He has forever been there … He was forever there.’ (Participant 6, female, 21 years old)

#### Re-briefing strengths through post-trauma thriver identity

Participants acknowledged that they were stronger as a result of the struggle with the CSA trauma, as illustrated in the following themes.

**Painful sharpening’s make a better pencil:** Participants 4 and 6 reflected on how their traumatic CSA ordeal shaped and sharpened their personal strengths intra- and interpersonally, as evidenced in the following comments:

‘… in life you go through painful sharpenings which will make you a better pencil … that’s me, I’ve been through challenges and this experience … and yes I’m not blunt … I’m sharp and I’m powered, and I’m a better pencil now … so me coming here was taking a sort of U-turn in life … to go back … so we can be the best pencil we can be, and to allow yourself to be guided by the handover to God in totality.’ (Participant 4, female, 22 years old)

‘And right now I can say I’m not thankful but I acknowledge what my perpetrator did to me, because if he hadn’t done that to me, I wouldn’t have realised my potential, I wouldn’t have blossomed into who I am today … and it’s because of him that I can say I’m a thriver, and there are many more to come.’ (Participant 6, female, 21 years old)

**Acknowledging the new ‘me’:** Participants 1 and 6 acknowledged that they see themselves as a new person through different lenses:

‘Whenever I hear that and I see myself that way, I look at myself through those lenses, those positive lenses, and I see a whole other person … and that’s the real me.’ (Participant 1, female, 30 years old)

‘It took a brave girl to try, it took the new me to see … now that I know and that I see, you will no longer control my life … this is my life to live and I know what’s best for me … you will try your way but this time I will not let you [*the abuse*] … I’m letting go of the fear of an unknown future for a known God … therefore, for all you have done to me, I forgive you.’ (Participant 6, female, 21 years old)

## Discussion

The reported resilience-enabling factors that assisted these participants in their journeys towards healing depict persistent processes of resisting the negative aftermath of the CSA, using both intrinsic and extrinsic resources as strengths, such as changing destructive to constructive rumination by being self-aware and engaging in cognitive reframing; utilising adaptive coping strengths; fostering meaning of the purpose of the trauma by engaging in religious activities and accessing social support in the safe holding environment of the homogeneous group context.

Several authors indicate that individuals who cope successfully after a traumatic event are committed to finding meaningful purpose in their lives, believing they can influence their surroundings, and in doing so learn and grow from their past experiences. This includes the active use of coping skills and relying on social support (Domhardt et al. 2015; Kaye-Tzadok & Davidson-Arad 2016; Marriott et al. 2013; Orbke & Smith 2013). These processes are important in restructuring one’s internal working model to positively recover from trauma. Furthermore, the healing group context and subsequent opportunities for witnessing other survivors’ healing journeys contributed towards alleviating the stigma, betrayal and powerlessness experienced by CSA survivors. This encouraged the women to realise their strengths and assets which were submerged beneath their pain, and subsequently shift from a victim to survivor narrative role.

The posttraumatic growth outcomes point to the wounded becoming the healer, a changed life philosophy, re-evaluating themselves and the hope of a future, faith as a deepened source of strength, and re-briefing their own strengths signified in their posttrauma identities. This transformational change appeared to promote successful adaptation and posttraumatic growth, rendering a shift from the survivor to thriver narrative role. This shift is maintained by assisting others, having a greater appreciation for their life opportunities, seeking comfort in their spirituality and ultimately seeing themselves with kinder eyes (Tedeschi & Calhoun 2004, 2006).

Although the authors regard the facilitators as expert companions (Tedeschi & Calhoun 2006) to be a valuable tool in the healing journey, the participants in this group did not reflect this. This could be because the witnessing in the group contributed to a personal identity from lone victim-child to collective powerful adult (Meekums 2000), and the facilitators were seen as an inherent part of this process, and not external drivers. Furthermore, instead of diagnosing deficits and prescribing treatment to address such deficits, the researchers as strengths-based facilitators assisted the survivors in identifying and building on their own strengths and resources. Thus, perhaps the process, rather than the facilitators, were responsible for the change that occurred.

## Limitations

The limitations of our findings are acknowledged. The dual role of the researchers as professional facilitators (expert companions) and researchers could be seen as a limitation. Furthermore, the findings are based on a small sample size. In addition, various contextual factors may have affected the outcomes of this study, such as the women being abused by a known perpetrator during middle childhood. Lastly, we acknowledge that the women’s inherent resilient processes and supportive ecologies or other unknown factors may have contributed to the observed growth (Walker-Williams & Fouché 2017).

## Conclusion and recommendations

The balanced S2T collaborative strengths-based group intervention programme, developed to enable resilience processes and facilitate posttraumatic growth outcomes in women survivors of CSA, appears to have been beneficial for the participants in this study. This study contributes to the body of evidence that now exists for strengths-based interventions for women survivors of CSA. The overall findings of this study suggest that it is important for helping professionals to incorporate a strengths-based approach into the recovery arsenal of CSA survivors without discounting the pathogenic nature of CSA. This can be done by incorporating a collaborative strengths-based approach, hereby partnering the traditional pathogenic and strengths-based approaches, and in doing so allowing helping professionals to bridge the gap between the hurt and pain caused by the CSA and the resilience processes and posttraumatic growth outcomes possessed by survivors of CSA. Further studies are required in this regard with a larger sample of CSA survivors.
